# Garcinol prevents oxidative stress-induced bone loss and dysfunction of BMSCs through NRF2-antioxidant signaling

**DOI:** 10.1038/s41420-024-01855-1

**Published:** 2024-02-16

**Authors:** Jilong Zou, Hongjun Chen, Xinming Fan, Zhenrui Qiu, Jiale Zhang, Jiabing Sun

**Affiliations:** Department of Orthopaedics, the First Affiliated Hospital of Harbin Medical University, Harbin, China

**Keywords:** Bone, Diseases

## Abstract

There are multiple published data showing that excessive oxidative stress contributes to bone loss and even bone tissue damage, and it is also correlated with the pathophysiology of bone degenerative diseases, including osteoporosis (OP). Garcinol, a polyisoprenylated benzophenone derivative, has been recently established as an anti-oxidant agent. However, it remains elusive whether Garcinol protects bone marrow mesenchymal stem cells (BMSCs) and bone tissue from oxidative stress-induced damage. Here, we explored the potential effects of Garcinol supplementation in ameliorating oxidative stimulation-induced dysfunction of BMSCs and bone loss in osteoporotic mice. In this study, we verified that Garcinol exerted potent protective functions in the hydrogen peroxide (H_2_O_2_)-induced excessive oxidative stress and dysfunction of BMSCs. Besides, Garcinol was also identified to improve the reduced bone mass and abnormal lineage commitment of BMSCs in the condition of OP by suppressing the oxidative stimulation. Subsequent analysis revealed that nuclear factor erythroid 2-related factor 2 (NRF2) might be a key regulator in the sheltering effects of Garcinol on the H_2_O_2_-regulated oxidative stress, and the protective functions of Garcinol was mediated by NRF2-antioxidant signaling. Collectively, Garcinol prevented oxidative stress-related BMSC damage and bone loss through the NRF2-antioxidant signaling, which suggested the promising therapeutic values of Garcinol in the treatment of oxidative stress-related bone loss. Therefore, Garcinol might contribute to treating OP.

## Introduction

Osteoporosis (OP), known as systemic bone metabolic disease, is primarily caused by insufficient bone formation. Nowadays, OP affects millions of patients globally, especially those after menopause and at old age [1, 2]. OP is topically accompanied by reduced bone mineral density (BMD), high risk in pathological fracture and deteriorated microstructure [3, 4]. Besides, OP is a global health concern and brings significantly increased economic burden to the society. As OP mainly occurs in populations aged 50 years old and above, the incidence of OP has been steadily raising, and the burden of OP is projected to be obviously larger due to the aging population globally [5, 6]. Therefore, developing preventable and treatable strategies is always the focus of OP treatment.

As we all know, the pathogenesis of OP is complex, and there are numerous risk factors resulting in bone loss and bone disorders, including aging and estrogen deficiency [7–9]. Recently, increasing evidences indicated that overproduced reactive oxygen species (ROS)-triggered oxidative stress is linked to the incidence and progression of OP [10–12]. In addition, the oxidative stress reduces the endogenous antioxidant defensive enzymes to induce dramatic damage to the cellular lipids, proteins, and nucleic acids, which is present throughout life [13–15]. Recently, multiple researches imply that oxidative stress, known as inducers of senescence, can induce bone loss and exert a negative role in bone remodeling and bone development [16]. For example, the sustained accumulation of oxidative stress is significantly increased in the condition of osteoporotic deterioration [17]. Besides, oxidative stress inhibits osteoblastic capacities, resulting in the impaired stemness of bone marrow mesenchymal stem cells (BMSCs). Generally, BMSCs are regarded as seed cells of osteoblasts and crucial components in the process of bone remodeling and thus play a pivotal role in the process of OP [18, 19]. Hence, blocking overproduced ROS and regulating oxidative stress are feasible strategies for OP treatment.

Nuclear factor erythroid 2-related factor 2 (NRF2) is recognized as a crucial and pivotal mediator of antioxidants [20, 21]. After oxidative stress stimulation, NRF2 transfers to the cell nucleus, binds to the antioxidant response element (ARE), and promotes the expression of antioxidant enzymes [21]. In addition, it was reported that Nrf2 deficiency led to the increase of ROS levels in mouse bone marrow cells, reduced trabecular BMD in femur, and then reduced cortical area of vertebrae [22]. Consequently, NRF2 might be an attractive candidate serving as a target of OP.

The antioxidant drugs exert obvious effects on the differentiation abilities and mineralization capacities of osteoblasts, and more importantly, they could be applied to ameliorate bone loss and OP [23, 24]. These evidences suggest that antioxidant drugs might be potentially used for the prevention of bone loss through counteracting the oxidative damage. Hence, natural antioxidants and its effective components have been subjects of increased research interest. Garcinol (camboginol), a well-known polyisoprenylated benzophenone, is mainly present in diverse Garcinia species. Garcinol has been immensely valued for its numerous medicinal values, such as antioxidant, acetyltransferase inhibitory, and anti-cancer effects [25, 26]. Recent researches have stated that Garcinol suppressed the receptor activator of nuclear factor (NF)-kB-ligand (RANKL)-caused osteoclastic formation by regulating MAPK and PI3K-AKT, which demonstrated that Garcinol could be applied as the focus in treating bone-related disorders [27]. However, the effects and molecular mechanisms of Garcinol in oxidative stress-induced bone loss need to be further investigated.

In this study, Garcinol, as an antioxidative drug, was revealed to attenuate the oxidative stress-induced BMSC dysfunction and bone loss. Based on this, the underlying mechanism and application potentials of Garcinol for OP therapy would be further verified in this research.

## Results

### Garcinol protects oxidative stress-induced dysfunction of BMSCs

**Fig. 1:**
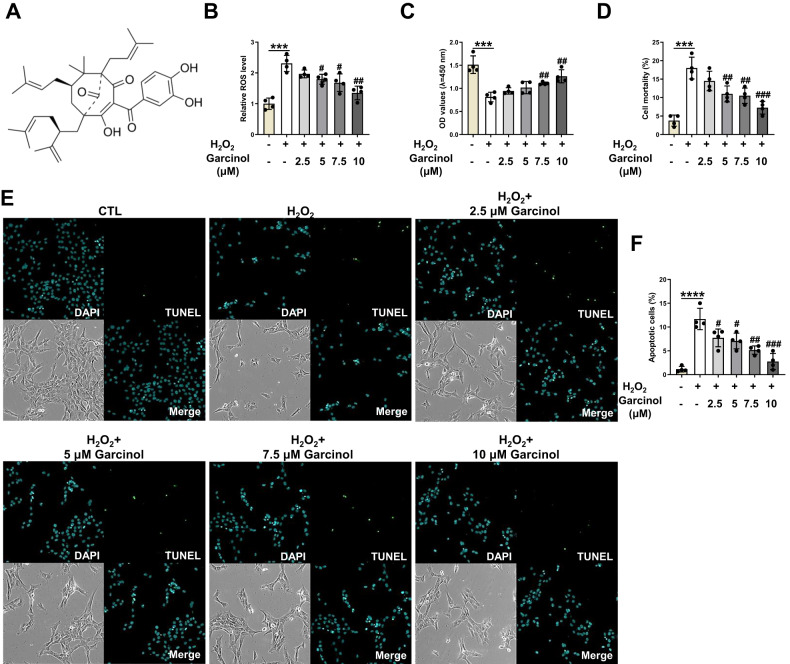
Garcinol treatment ameliorates H_2_O_2_-caused injury in BMSCs. **A** The chemical structure of Garcinol. **B** Analysis of ROS level was conducted to examine the ROS accumulation of BMSCs. **C** CCK-8 assay was utilized to assess the cell viability of BMSCs. **D** Trypan blue exclusion assay was carried out to measure the cell death of BMSCs. **E** The apoptosis of BMSCs was observed by TUNEL staining. **F** The quantitative analysis of TUNEL staining. *N* = 4, the comparisons among groups were analyzed by ANOVA analysis. ***P < 0.001 and ****P < 0.0001 vs. the CTL group. #*P* < 0.05, ##*P* < 0.01 and ###*P* < 0.001 vs. the H_2_O_2_ group. H_2_O_2_ hydrogen peroxide, ROS reactive oxygen species.

Based on previous reports, hydrogen peroxide (H_2_O_2_) was widely applied to induce oxidative stress in different cells [28–32]. Hence, BMSCs were pretreated with H_2_O_2_ at 100 μM for 2 h to establish the oxidative stress-treated cell models, and the establishment of this model laid the groundwork for further analysis of the Garcinol functions. Some reports have speculated that oxidative stimulation might be responsible for regulating the biological functions of BMSCs. Thus, we then investigated whether different concentrations of Garcinol could inhibit the elevated ROS level in H_2_O_2_ stimulation-induced BMSCs. The results reflected that H_2_O_2_ treatment effectively activated ROS production in BMSCs, which was gradually reversed by Garcinol (Fig. 1A, B). Besides, 10 μm Garcinol exhibited the best effect among all drug intervention group (Fig. 1B).

To fully depict the specific effects of Garcinol on ROS-mediated injury, the role of Garcinol in the viability and survival of BMSCs was detected first. We investigated the major roles of various concentrations of Garcinol in the cell viability of BMSCs after treatment with H_2_O_2_ at 100 μM for 2 h. As indicated in Fig. 1C, the cell viability of BMSCs was notably descend after H_2_O_2_ administration, while it was gradually improved after the treatment of Garcinol from 2.5 to 10 μM (Fig. 1C). Notably, the protective role of Garcinol was the best when the concentration reached 10 μM (Fig. 1C). The trypan blue exclusion assay was further carried out to explore the roles of Garcinol in the ROS-induced cell death. The results validated that Garcinol treatment decreased the cell mortality in H_2_O_2_-induced BMSCs in a concentration-dependent manner (Fig. 1D). Then, the protection of Garcinol against oxidative stress-caused apoptosis of BMSCs were assessed. The outcomes of TUNEL staining denoted that Garcinol at 2.5, 5, 7.5, 10 μM obviously diminished the percentage of apoptotic BMSCs induced by H_2_O_2_ treatment, indicating that H_2_O_2_-induced cell death might be mainly caused by apoptosis, and it could be mitigated by Garcinol treatment (Fig. 1E, F). All in all, Garcinol was proved to alleviate the oxidative stress-reduced cell viability, cell death and apoptosis of BMSCs. Therefore, Garcinol was able to reverse oxidative stress-induced BMSC injury and restore the dysfunctions of BMSCs.

### Garcinol alleviates oxidative stress-caused abnormal fate determination of BMSCs

**Fig. 2:**
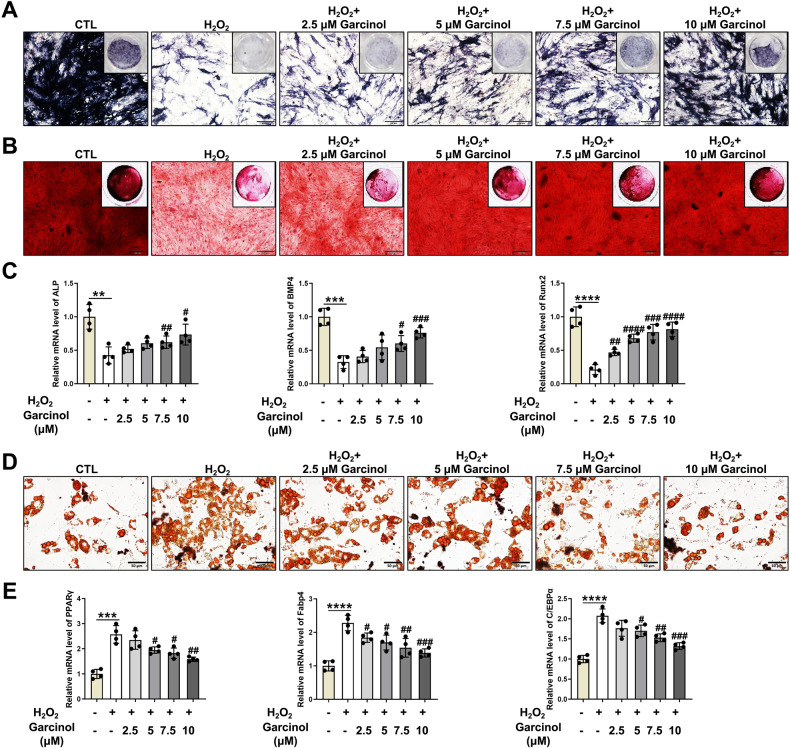
Garcinol treatment restores H_2_O_2_-induced abnormal cell fate of BMSCs. **A** The osteoblastic commitment of H_2_O_2_-treated BMSCs after Garcinol administration was determined by ALP staining. Scale bar=100 μm. **B** The mineralized depositions of BMSCs was measured by ARS staining. Scale bar=100 μm. **C** Measurements of mRNA levels of osteoblastic marker genes in BMSCs. **D** The adipogeneic differentiation of H_2_O_2_-treated BMSCs after Garcinol supplementation was quantified by ORO staining. Scale bar=50 μm. **E** The mRNA levels of key adipocyte-related genes were evaluated by qRT-PCR analysis. *N* = 4. The comparisons among groups were analyzed by ANOVA analysis. **P < 0.01, ***P < 0.001,  and ****P < 0.0001 vs. the CTL group. #*P* < 0.05, ##*P* < 0.01 ###*P* < 0.001 and ####*P* < 0.0001 vs. the H_2_O_2_ group. H_2_O_2_ hydrogen peroxide, ALP alkaline phosphatase, ARS alizarin red S, ORO oil red O, BMP4 bone morphogenetic protein 4, Runx2 runtrelated transcription factor 2, PPARγ peroxisome proliferator-activated receptor γ, Fabp4 fatty-acid-binding protein 4, C/EBPα CCAAT enhancer-binding protein α.

In order to characterize the specific functions of Garcinol in H_2_O_2_-induced abnormal fate of BMSCs, H_2_O_2_ was applied to induce intracellular oxidative stress in BMSCs, and Garcinol from 2.5 to 10 μM was applied to treat BMSCs. The capacity of osteogenesis, which was indicated by calcified nodules, was identified by alkaline phosphatase (ALP) and alizarin red S (ARS) staining (Fig. 2A, B). The results of ALP and ARS staining indicated that the number and area of mineralized nodules of BMSCs were markedly decreased after H_2_O_2_ stimulation, while gradually elevated upon different concentrations of Garcinol treatment (Fig. 2A, B). Furthermore, compared with H_2_O_2_ group, the mRNA levels of the well-known osteoblast-specific genes, including ALP, bone morphogenetic protein 4 (BMP4) and runt-related transcription factor 2 (Runx2), were obviously increased in the Garcinol group (Fig. 2C). Considering these results, we supposed that Garcinol restored the oxidative stress-caused weakened osteogenic differentiation of BMSCs.

To elucidate the possible functions of Garcinol in the adipogenesis, BMSCs were separately treated by H_2_O_2_ and varying concentrations of Garcinol, then cultured in the adipogenesis differentiation medium. The analysis of oil red O (ORO) staining demonstrated that Garcinol retarded H_2_O_2_-stimulated facilitation in the lipid droplet formation of BMSCs (Fig. 2D). Consistently, the results were also comfirmed by qRT-PCR assay. As depicted in Fig. 2E, the levels of critical markers of adipocyte differentiation, including peroxisome proliferator-activated receptor γ (PPARγ), fatty-acid-binding protein 4 (Fabp4) and CCAAT enhancer-binding protein α (C/EBPα), were significantly heightened by H_2_O_2_-caused oxidative stress, while markedly blocked by Garcinol treatment (Fig. 2E). The above findings indicated that Garcinol rescued the H_2_O_2_-induced abnormal differentiation tendency of BMSCs.

### Garcinol restores the oxidative stress and bone loss in osteoporotic mice

**Fig. 3:**
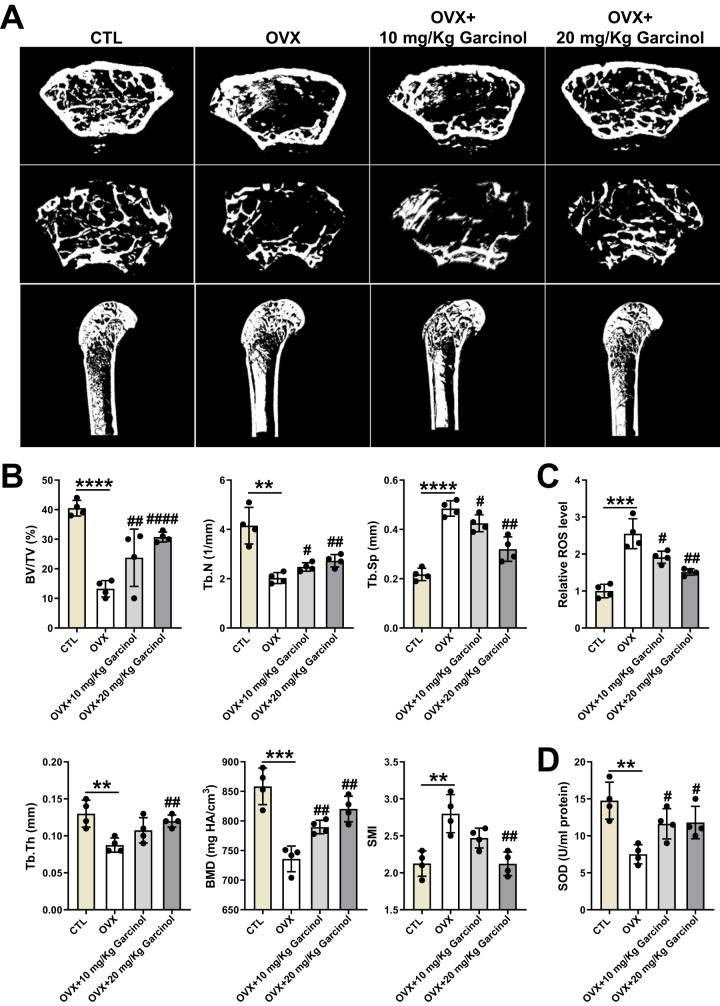
The prevention of Garcinol in oxidative stress-related bone loss in osteoporotic mice. **A** The representative μCT images of mouse distal femurs from the CTL group, OVX group, OVX + 10 mg/Kg Garcinol group and OVX + 20 mg/Kg Garcinol group. **B** The trabecular structural parameters of mice were evaluated. **C** Garcinol inhibited the ROS level in BMSCs from osteoporotic mice. **D** Garcinol elevated the SOD level in BMSCs from OVX mice. *N* = 4. The comparisons among groups were analyzed by ANOVA analysis. **P < 0.01, ***P < 0.001 and ****P < 0.0001 *vs*. the CTL group. #*P* < 0.05, ##*P* < 0.01 and ####*P* < 0.0001 vs. the OVX group. μCT microcomputed tomography, BV/TV bone volume to tissue volume, Tb.N trabecular number, Tb.Sp trabecular separation, Tb.Th trabecular thickness, BMD bone mineral density, SMI structure model index, ROS reactive oxygen species, SOD superoxide dismutase.

OP has been recognized of being always accompanied with increased oxidative stress and accumulation of ROS [33–36]. Thus, the bilateral ovariectomy (OVX) mouse models were utilized to estimate the protective effect of Garcinol on OP and oxidative stress-induced injury in vivo. According to subsequent analysis, OVX mice displayed significant damaged architecture of trabecular bones in the distal femurs, confirming that there was an obvious reduction in bone mass in OVX-related osteoporotic mouse models (Fig. 3A). Moreover, Garcinol-treated OVX mice also showed evident improvements in architecture of trabecular bones in the distal femurs in osteoporotic mice, indicating that Garcinol restored the bone loss in osteoporotic mice (Fig. 3A). Similarly, further analysis of microcomputed tomography (μCT) scanning indicated that BV/TV, Tb.N, Tb.Th and BMD were evidently lessened, accompanied by increased Tb.SP and structure model index (SMI) in ovariectomized mice relative to the CTL group (Fig. 3B). Furthermore, compared with OVX group, a higher BV/TV, Tb.N, Tb.Th and BMD and a lower Tb.SP and SMI could be observed in OVX mice after Garcinol treatment, suggesting that Garcinol effectively restored the OVX-induced deterioration of microstructure in osteoporotic mice (Fig. 3B).

**Fig. 4:**
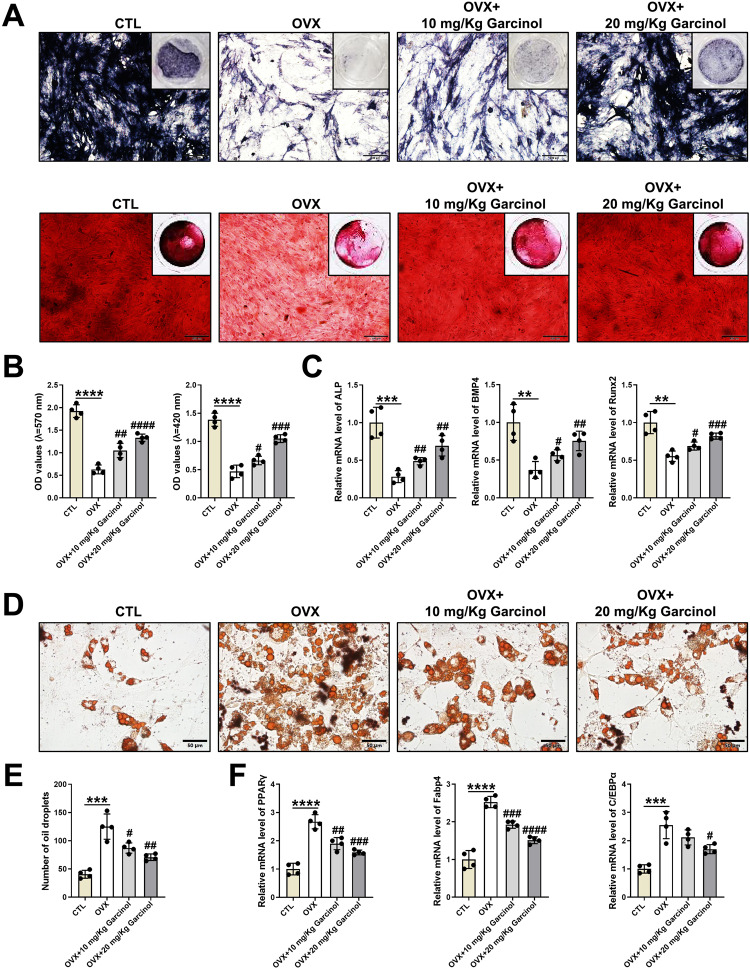
The protective roles of Garcinol in abnormal differentiation potentials of BMSCs from osteoporotic mice. **A** ALP and ARS staining images of BMSCs after osteogenic induction, which were isolated from the CTL group, OVX group, OVX + 10 mg/Kg Garcinol group and OVX + 20 mg/Kg Garcinol group. Scale bar=100 μm. **B** The statistical results of ARS staining (left) and ALP staining (right). **C** The expression levels of ALP, BMP4 and Runx2 were calculated by qRT-PCR analysis. **D** ORO staining images of BMSCs after adipogenic differentiation induction. Scale bar=50 μm. **E** The statistical results of ORO staining. **F** The mRNA expression of PPARγ, Fabp4 and C/EBPα was assessed by utilizing qRT-PCR analysis. *N* = 4. The comparisons among groups were analyzed by ANOVA analysiss. **P < 0.01, ***P < 0.001 and ****P < 0.0001 vs. the CTL group. #*P* < 0.05, ##*P* < 0.01, ###*P* < 0.001 and ####*P* < 0.0001 vs. the OVX group. ARS alizarin red S, ALP alkaline phosphatase, BMP4 bone morphogenetic protein 4, Runx2 runt-related transcription factor 2, ORO oil red O, PPARγ peroxisome proliferator-activated receptor γ, Fabp4 fatty-acid-binding protein 4, C/EBPα CCAAT enhancer-binding protein α.

Additional analysis of ROS revealed that, compared with the BMSCs from the CTL group, the OVX group exhibited a markedly elevated ROS level, which indicated a remarkable oxidative capability (Fig. 3C). However, the increased ROS level was effectively mitigated in the OVX mice administered with Garcinol (Fig. 3C). Besides, in contrast to CTL group, the BMSCs from OVX group showed an obvious reduction in superoxide dismutase (SOD) activity, that represented the antioxidative ability (Fig. 4D). Interestingly, the decreased SOD level in osteoporotic mice were partially restored by Garcinol administration, implying that Garcinol treatment could block the elevated oxidative stress level in BMSCs from OP (Fig. 4D). The above findings demonstrated that Garcinol restored the oxidative stress-related bone loss in mice with OP.

### Garcinol attenuates the abnormal lineage commitment of BMSCs in osteoporotic mice

Based on the fact that OP-impaired bone-fat balance might be strongly correlated with oxidative stimulation, we detected the roles of Garcinol in cell fate of BMSCs under osteoporotic conditions. Next, to further gain insight into the beneficial functions of Garcinol in OP, the BMSCs of mice from different groups were isolated, respectively. Then, the fate shift between adipocytes and osteoblasts of BMSCs were determined. As exhibited in Fig. 4A, B, the matrix mineralization of BMSCs was markedly less in OVX-caused osteoporotic mice than CTL group (Fig. 4A, B). In addition, compared with OVX group, a significant higher osteoblastic ability could be observed in BMSCs from OVX + 10 mg/Kg Garcinol group and OVX + 20 mg/Kg Garcinol group (Fig. 4A, B). In addition, the relative mRNA levels of important osteoblastic markers, including ALP, BMP4 and Runx2, were statistically decreased in BMSCs derived from OVX group, while the decreased expression levels of these genes were upregulated by Garcinol supplementation (Fig. 4C). Furthermore, the adipogenesis of BMSCs after stimulation with different treatment was further estimated through additional experiments. As a result, the formation of oil droplets was significantly promoted in BMSCs derived from the OVX group (Fig. 4D, E). After Garcinol treatment, the enhanced adipogenic abilities of BMSCs from OVX mice was abolished, suggesting that the adipocyte formation of BMSCs in osteoporotic mice could be hampered by Garcinol (Fig. 4D, E). Furthermore, the mRNA expression of adipocyte specific genes was found to be higher in BMSCs from OVX mice than that from CTL group (Fig. 4F). However, Garcinol treatment decreased the expression of PPARγ, Fabp4 and C/EBPα in the BMSCs from OVX + 10 mg/Kg Garcinol group and OVX + 20 mg/Kg Garcinol group (Fig. 4F). Overall, Garcinol might restore the bone loss by repairing the disturbed differentiation of BMSCs from oxidative stress-related OP.

### NRF2 may act as a key regulator in the process of Garcinol improving H_2_O_2_-induced oxidative stress

**Fig. 5:**
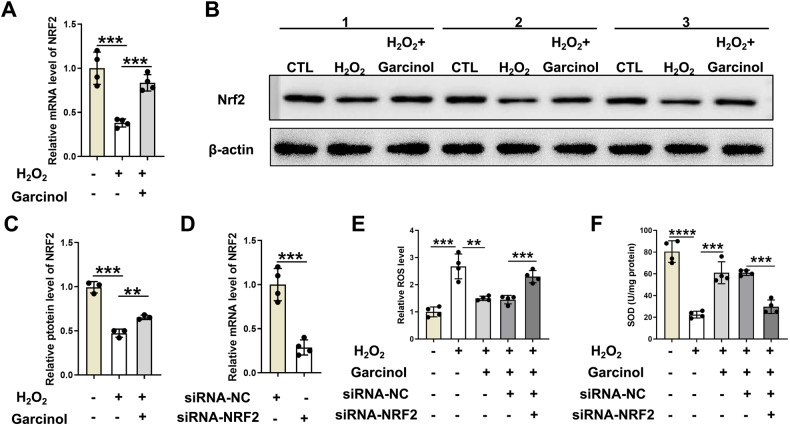
The roles of NRF2 in Garcinol-mediated protection from the H_2_O_2_-induced oxidative stress. **A** The mRNA expression of NRF2 was analyzed by qRT-PCR analysis. **B** The relative protein levels of NRF2 were measured by western blot assay. **C** The quantitative analysis of western blot assay. **D** The relative expression of NRF2 was detected in BMSCs after transfection of siRNA-NRF2. **E** The relative ROS level of BMSCs was observed. **F** The level of SOD in BMSCs was evaluated. *N* = 4, The comparisons among groups were analyzed by ANOVA analysis. **P < 0.01, ***P < 0.001 and ****P < 0.0001. NRF2 nuclear factor erythroid 2-related factor 2, ROS reactive oxygen species, SOD superoxide dismutase.

Subsequently, an exploration was performed on the potential mechanisms of Garcinol in ameliorating oxidative stress-contributed injury of BMSCs. Given the important roles of NRF2 in preventing oxidative stress-induced damage, NRF2 was selected for additional analysis. The BMSCs were incubated with H_2_O_2_ at 100 μM and Garcinol at 10 μM, respectively, and the relative mRNA expression of NRF2 in BMSCs were evaluated. As displayed in Fig. 5A, the relative expression level of NRF2 was significantly down-regulated in BMSCs after H_2_O_2_ administration, while it was reversed by Garcinol treatment (Fig. 5A). Furthermore, the protein levels of NRF2 were notably declined in BMSCs after H_2_O_2_ supplementation, but the declined NRF2 protein level was partly recovered by Garcinol treatment (Fig. 5B, C).

Considering NRF2 might be a crucial regulator in mediating the protections of Garcinol in the H_2_O_2_-induced oxidative stress, the expression of NRF2 was significantly decreased after knockdown of NRF2 by using siRNA-NRF2 in BMSCs, and we further tested the role of NRF2 in H_2_O_2_-induced oxidative stress of BMSCs (Fig. 5D). The results of intracellular ROS measurement assays expounded that Garcinol could obviously inhibit H_2_O_2_ treatment-stimulated elevated ROS level in BMSCs, while the impact was blocked after transfection with siRNA-NRF2 (Fig. 5E). Additional experiments determined the importance of NRF2 in the functions of Garcinol against H_2_O_2_-induced oxidative stress (Fig. 5F). The results of the SOD detection assay revealed that knockdown of NRF2 appeared to reverse the inhibitory ability of Garcinol in H_2_O_2_-caused oxidative stress (Fig. 5F). Hence, we hypothesized that NRF2 could regulate the protective effects of Garcinol on the oxidative stress-induced damaged BMSCs.

### Garcinol possesses the protective activities against oxidative stress via NRF2-antioxidant signaling

**Fig. 6:**
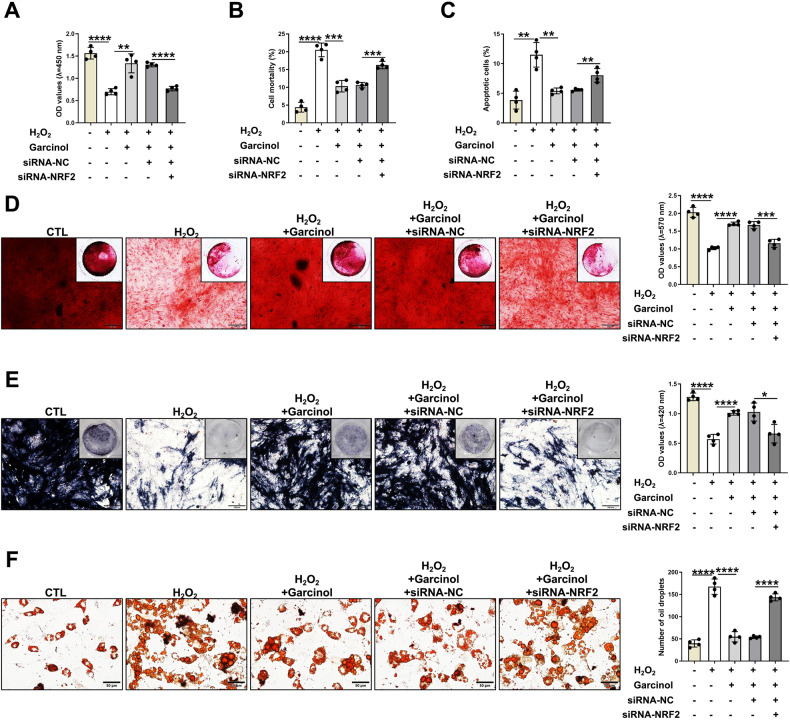
Garcinol protects damaged BMSCs and bone loss from oxidative stress by activating NRF2-antioxidant signaling. **A** The cell viability of BMSCs from CTL group, H_2_O_2_ group, H_2_O_2_+Garcinol group, H_2_O_2_+Garcinol+siRNA-NC, H_2_O_2_+Garcinol+siRNA-NRF2 group, was confirmed by CCK-8. **B** Trypan blue exclusion assay was utilized to quantify the cell death of BMSCs from CTL group, H_2_O_2_ group, H_2_O_2_+Garcinol group, H_2_O_2_+Garcinol+siRNA-NC, H_2_O_2_+Garcinol+siRNA-NRF2 group. **C** The quantification of TUNEL staining. **D** ARS staining and quantitative analysis. Scale bar=100 μm. **E** ALP staining and quantity of mineralization. Scale bar=100 μm. **F** ORO staining and quantitative analysis of adipocytes. Scale bar=50 μm. *N* = 4, The comparisons among groups were analyzed by ANOVA analysis. *P < 0.05, **P < 0.01, ***P < 0.001 and ****P < 0.0001. H_2_O_2_ hydrogen peroxide, NRF2 nuclear factor erythroid 2-related factor 2, ARS alizarin red S, ALP alkaline phosphatase.

To further confirm the crucial role of NRF2 in the inhibitory effects of Garcinol in H_2_O_2_-induced oxidative stress and abnormal functions of BMSCs, the biological functions and lineage commitment of BMSCs were then tested. The BMSCs were supplemented with H_2_O_2_ at 100 μM and Garcinol at 10 μM, respectively, and then transfected with siRNA-NRF2 for subsequent experiments. The data from CCK-8 assay displayed that the diminished cell viability caused by H_2_O_2_ treatment was recovered by Garcinol, and such effect could be blocked by knock-down of NRF2 (Fig. 6A). Based on the outcomes of trypan blue exclusion assay, compared with H_2_O_2_ group, the cell death was reduced in the BMSCs from H_2_O_2_+Garcinol group while apparently raised in the H_2_O_2_+Garcinol+siRNA-NRF2 group, suggesting that NRF2 was indispensable for the protection of Garcinol against H_2_O_2_-induced cell death. (Fig. 6B). Besides, the qualitative analysis of TUNEL staining indicated that the increased percentage of apoptotic BMSCs induced by H_2_O_2_ was attenuated after Garcinol treatment, while it was remarkably reversed by silencing NRF2 (Fig. 6C).

Next, ARS, ALP staining and ORO staining were carried out to assess the functions of NRF2 in Garcinol-mediated specific effects in the H_2_O_2_-induced abnormal fate of BMSCs. On basis of the staining results, Garcinol contributed to an apparent protection in the osteogenic-adipogenic imbalance of BMSCs caused by H_2_O_2_-induced oxidative stress, while the protective ability was partly blocked by deletion of NRF2 (Fig. 6D–F). Such findings provided important evidence for the importance of NRF2 in the protective activity of Garcinol in the oxidative stress-induced abnormal cell fate of BMSCs (Fig. 6D–F). Collectively, Garcinol exerted protective effects against oxidative stress-induced abnormal functions of BMSCs via the NRF2-antioxidant signaling.

## Discussion

Garcinol, a polyisoprenylated benzophenone, is well-known as an antioxidant [37–39]. Chang et al. illustrated that Garcinol had the potentials to prevent and attenuate dysfunction of cardiomyocytes through suppressing the apoptosis and inflammation [40]. Kim et al. revealed that Garcinol efficiently attenuated the excessive oxidative stress and apoptosis in cisplatin-treated tubular epithelial cells [41]. In this study, the functions of Garcinol in oxidative stress-induced injury of BMSCs were investigated through establishing the models of H_2_O_2_-stimulated BMSCs. The investigation results revealed that administration of Garcinol ameliorated H_2_O_2_-caused reduction in cell viability, increase in cell death and apoptosis of BMSCs by inhibiting excessive oxidative stress, indicating the protective functions of Garcinol in H_2_O_2_-associated dysfunction of BMSCs. Our research further supported the prevention of Garcinol in the H_2_O_2_-caused imbalanced fate shift of BMSCs between osteoblasts and adipocytes. Also, administration of Garcinol could restored the abnormal cell fate of BMSCs caused by excessive oxidative stress. As now, our research first revealed the protective roles of Garcinol in oxidative stress-caused injury and dysfunction of BMSCs.

Oxidative stress participates in the progression of OP, including impaired bone remodeling, bone ageing and deterioration of microstructure [42–44]. Antioxidants have been broadly considered as potent agents for bone loss and OP [45–47]. Therefore, we focused on antioxidants and determined the protective functions of Garcinol in excessive oxidative stress and bone tissue damage. In our study, excessive oxidative stress could be observed in osteoporotic mice, implying that excessive oxidative stress was closely involved in the pathogenesis of OP. Additional analysis supported that supplementation of Garcinol not only restored the abnormal cell fate decision of BMSCs but also rescued the osteoporotic phenotype in the osteoporotic mice by inhibiting excessive oxidative stress. To date, we first focused on the functions of Garcinol on excessive oxidative stress-associated dysfunctions of BMSCs and bone loss, laying the foundation for the future utilization of Garcinol to relieve oxidative stress-related bone tissue damage in osteoporotic patients.

What’s more, we also demonstrated an in-depth mechanism for inhibition of excessive oxidative stress by Garcinol. Our study revealed that Garcinol might exert an antioxidant role in H_2_O_2_-related injury of BMSCs and osteoporotic mice through a vital regulator NRF2. NRF2, a master transcription factor for stress response, possesses the ability to manipulate the cellular defense against toxic and oxidative insults by modulating the expression level of antioxidant and detoxification genes [48–50]. Mechanistically, to explore the role of NRF2 in Garcinol-mediated protective roles, BMSCs were transfected with siRNA-NRF2 to reduce the level of NRF2. The results indicated that knockdown of NRF2 appeared to deteriorate the inhibitory ability of Garcinol in the H_2_O_2_-caused oxidative stress. According to these results, we highlighted the importance of NRF2 in the protection function of Garcinol in the H_2_O_2_-induced oxidative stress. Furthermore, this research also exhibited that Garcinol exerted protective effects against oxidative stress-induced dysfunction of BMSCs and bone loss via the NRF2-antioxidant signaling. This study has elucidated the importance of NRF2 in Garcinol-mediated protective functions in excessive oxidative stress-induced injury for the first time.

Although there were some novel findings in our study, it needs to be acknowledged that there are also certain limitations. Firstly, the current study has not fully explained the detailed mechanism of NRF2 in Garcinol-mediated protection against oxidative stress. Secondly, the impact of Garcinol on the downstream targets of NRF2 is also unclear. Furthermore, the effect of Garcinol on NRF2 was not assessed in vivo. Further research and exploration are still required to solve these issues. In addition, our study probed into the functions of Garcinol based on restoring excessive oxidative stress-induced dysfunction and abnormal cell fate of BMSCs, however, the role of Garcinol in excessive oxidative stress-induced bone formation and bone resorption remains to be determined. Future functional investigation will explore the anti-oxidative stress effect of Garcinol on osteoblasts. Moreover, we did not evaluated the toxicity and side effects of Garcinol to BMSCs or mice in vitro and in vivo. Hence, the focus of future observation is to investigate the toxicity and side effects of Garcinol.

**Fig. 7:**
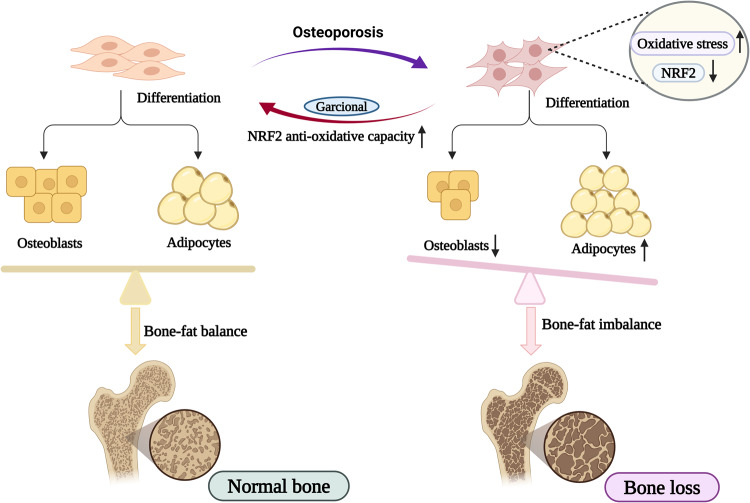
A schematic diagram illustrating the protective effects and potential mechanism of Garcinol on oxidative stress-induced damaged BMSCs and bone loss. Osteoporosis always accompanies with increased oxidative stress and accumulation of ROS. The increased oxidative stress led to the abnormal functions and imbalance between adipogenesis and adipogenesis of BMSCs, and even induced bone loss. Garcinol supplementation restored the oxidative stress-induced dysfunctions of BMSCs and bone loss through activating NRF2-antioxidant signaling.

To sum up, our research revealed that Garcinol could prevent oxidative stress-induced dysfunction and abnormal cell fate of BMSCs, and subsequently ameliorate bone disorders in osteoporotic mice via activating NRF2-antioxidant signaling (Fig. 7). This study provided convincing evidence that Garcinol might be developed as a potent and promising candidate medicine in the treatment of OP and other bone disorders correlated with oxidative stress in the future. The eventual application of Garcinol in osteoporotic patients is of great significance and worthy of further clinical investigation.

## Materials and methods

### Cell isolation and cell culture

The mouse BMSCs were separated from the bone marrows of bilateral femur in 8-week-old C57BL/6 J mice in accordance with the previous study [51]. BMSCs were maintained in BMSC culture medium (Cyagen Biosciences, China) and placed in a standard culture environment. The fresh culture medium was replaced every other day. When the final density of BMSCs was closed to 90%, the cells were digested under the action of 0.25% trypsin (Cyagen Biosciences, China) and then sub-cultured for further analysis.

### Cell treatment

BMSCs were treated with H_2_O_2_ (Sigma-Aldrich, USA) at 100 μM for 2 h to induce the intracellular oxidative stress [28, 52, 53], followed by treatment with Garcinol (2.5, 5, 7.5, 10 μM) for 48 h.

### Intracellular ROS measurement

The intracellular ROS levels of BMSCs were determined according to the previous reports [54]. In brief, the BMSCs were washed three times using PBS buffer and then incubated with the diluted DCFH-DA fluorescent probe at 37 °C in the dark for 20 min. Next, the cells were rinsed by FBS-free cell culture medium three times. Finally, the absorbance at 488 and 525 nm was analyzed through a SpectraMax EM Microplate Reader (Molecular Devices, USA), followed by the calculation and analysis of the ROS levels.

### Detection of cell viability

The CCK-8 assays were carried out to identify the cell viability in BMSCs. Briefly, BMSCs were seeded in 96-well plates (Nest, China) at a final density of 5000/well. When the cell confluence reached 90%, the culture medium was added with Garcinol or H_2_O_2_, and the cells were pre-incubated with 10-μL CCK-8 solution (Tongren, Japan). After 4 h, the optical density (OD) of cells were identified through a microplate reader (TECAN, Switzerland) at 450 nm.

### Trypan blue exclusion assay

The death of BMSCs was estimated by trypan blue exclusion assay. Firstly, BMSCs were seeded in six-well plates and cultured for one day. After that, the cells were detached in the presence of EDTA solution (Beyotime Biotechnology, China) and then maintained in trypan blue solution. After 3-5 min of staining, the number of live and dead BMSCs was automatically analyzed using an automated cell counter (Thermo, USA).

### Apoptosis assay

The apoptotic BMSCs were analyzed utilizing an in-situ Cell Death Detection Kit (Roche, USA) based on the manufacturer’s manuals. The TUNEL-positive cells were viewed through a fluorescence microscope (Nikon, Japan), and the pictures of apoptotic BMSCs were captured.

### Alkaline phosphatase (ALP) staining

The osteoblastic formation and mineralization abilities of BMSCs after osteogenesis for 7 days were detected by ALP staining. To assess the osteoblastic formation, the BMSCs seeded in 24-well plates (Nest, China) were continuously maintained in osteogenic induction medium (Cyagen Biosciences, China) under differentiating conditions. After osteogenesis for continuous 7 days, the mineralization of the calcium nodules was observed through ALP staining by using ALP staining kits (CTCC Bioscience, China). The stained BMSCs were subsequently visualized using an invert light microscope (Nikon, Japan), followed by the capture of representative images. To visualize the osteoblastic activity of BMSCs, the stained mineralization was dissolved by 10 mM p-nitrophenyl phosphate (Thermo, USA), and then the OD values at 420 nm were measured and calculated.

### Alizarin Red S (ARS) staining

The accumulation of calcium deposition of BMSCs after osteogenesis for 14 days was ascertained via ARS staining. Specifically, the cells were fixed in 4% PFA then incubated with ARS staining kits (Cyagen Biosciences, China) for about 30 min. Next, the cells were rinsed by PBS and visualized under an invert light microscope (Nikon, Japan), and the representative images were captured. To quantify the calcium deposition, the stained mineralized bone nodules were dissolved using 10% cetylpyridinium chloride (Sigma, USA), and the OD values were visually seen at 570 nm under a microplate reader (TECAN, Switzerland).

### Quantitative real-time polymerase chain reaction (qRT-PCR)

TRIzol reagents were used for extracting the total RNAs from BMSCs, and the complementary DNAs (cDNAs) were synthesized through PrimeScript RT reagent Kits (Takara, Japan). The amplification of cDNAs was routinely performed by qRT-PCR analysis using SYBRGreen Mix (Takara, Japan). The GAPDH was considered as a housekeeping gene for measuring the expression of target genes by the comparative 2^−△△Ct^ method. The primer sequences were listed in Table 1.

**Table 1:** The sequences of primers for qRT-PCR analysis.

Genes		Primer sequences (5’ to 3’)
ALP	F	ACAACCTGACTGACCCTTCG
	R	TCATGATGTCCGTGGTCAAT
BMP4	F	TCGTTACCTCAAGGGAGTGG
	R	ATGCTTGGGACTACGTTTGG
Runx2	F	AGAAGGCACAGACAGAAGCTTGA
	R	AGGAATGCGCCCTAAATCACT
PPARγ	F	TCACAAGAGGTGACCCAATG
	R	CCATCCTTCACAAGCATGAA
Fabp4	F	TTCCTGTCGTCTGCGGTGATT
	R	GATGCCTTTGTGGGAACCTGG
C/EBPα	F	GTGTGCACGTCTATGCTAAACCA
	R	GCCGTTAGTGAAGAGTCTCAGTTTG
GAPDH	F	CATCACTGCCACCCAGAAGAC
	R	CCAGTGAGCTTCCCGTTCAG

### Oil Red O (ORO) staining

ORO staining was conducted to identify the adipogenic committed cells. Briefly, the BMSCs were fixed using 4% PFA solution, dyed with ORO solution (Cyagen Biosciences, China) for 30 min, and then observed through an inverted light microscopy (Nikon, Japan). Finally, the representative images were acquired and the number of adipocytes was obtained.

### Animal experiments

A total of 40 female C56BL/6J mice aging 6–8 weeks were provided by Beijing Vital River Laboratory Animal Technology Co., Ltd. The mice were randomly divided into following four groups: CTL group, OVX group, OVX + 10 mg/Kg Garcinol group and OVX + 20 mg/Kg Garcinol group. After an acclimatization period of one week, the mice in OVX group underwent bilateral ovariectomy (OVX) according to a previous study [4]. While, the mice in CTL group were subjected to sham operation. The osteoporotic mouse models received drug treatment four weeks after surgery.

The mice from OVX + 10 mg/Kg Garcinol group and OVX + 20 mg/Kg Garcinol group were given intraperitoneal injection of Garcinol (10 mg/Kg or 20 mg/Kg) for 14 consecutive days [26, 55–57]. The mice in CTL and OVX group were intraperitoneally injected with normal saline. After completion of experiments, the femurs of mice were obtained and subject to μCT analysis. The trabecular structural parameters and SMI were analyzed. All procedures were approved by the Ethics Committee of the First Affiliated Hospital of Harbin Medical University (2022071).

### Measurement of oxidative stress

The level of MDA was separately analyzed through colorimetric/fluorometric assay kits (Sigma, USA) as instructed by the manufacturers.

### Cell transfection

To generate NRF2 knock-down cells, the BMSCs were transfected with short interfering RNAs (siRNAs) targeting Nrf2 (siRNA-NRF2) or negative controls (siRNA-NC, Riobio, Guangzhou) by Lipofectamine 2000 reagents (Invitrogen, USA) following the manufacturer’s instructions.

### Western blot (WB) assay

The cells were obtained and lysed in RIPA buffer (Beyotime, China) at 4 °C. Western blot assays were carried out as mentioned previously [58]. The following antibodies used in the present study were obtained from Abcam (NRF2) and Cell Signaling Technology (β-actin). The immuno-reactive bands were analyzed by enhanced chemiluminescence (ECL) and then calculated by Image Software (NIH, USA).

### Statistical analysis

The data were shown as mean ± standard deviation (SD). The statistical analysis was conducted by SPSS 20.0 software (SPSS, Chicago, USA). Analyses were performed by one-way ANOVA or Student’s *t*-test. *P* < 0.05 indicated a significant difference.

### Supplementary information

Original Data File

## Data Availability

The datasets generated and analyzed during the current study are available from the corresponding author upon reasonable request.
