# Supplementary figures and images for: Garcinol prevents oxidative stress-induced bone loss and dysfunction of BMSCs through NRF2-antioxidant signaling
Source: Cell Death Discov. 2024 Feb 16;10:82. doi: 10.1038/s41420-024-01855-1 (PMC10873372; doi:10.1038/s41420-024-01855-1)

Figure 5B


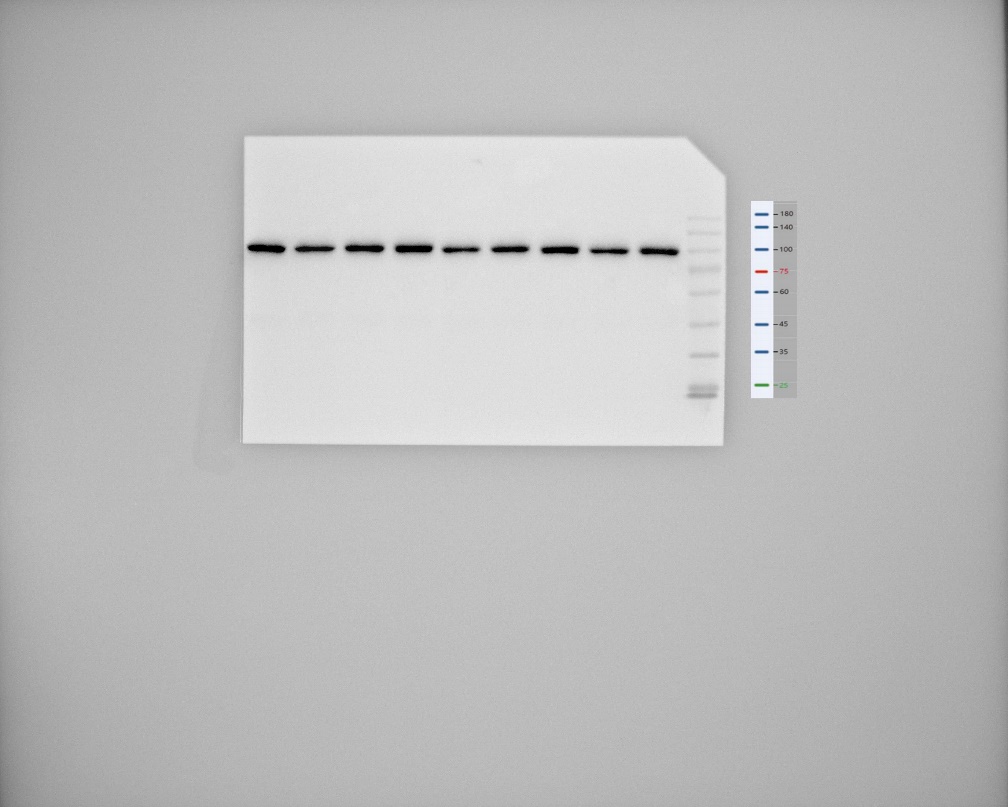


Nrf2


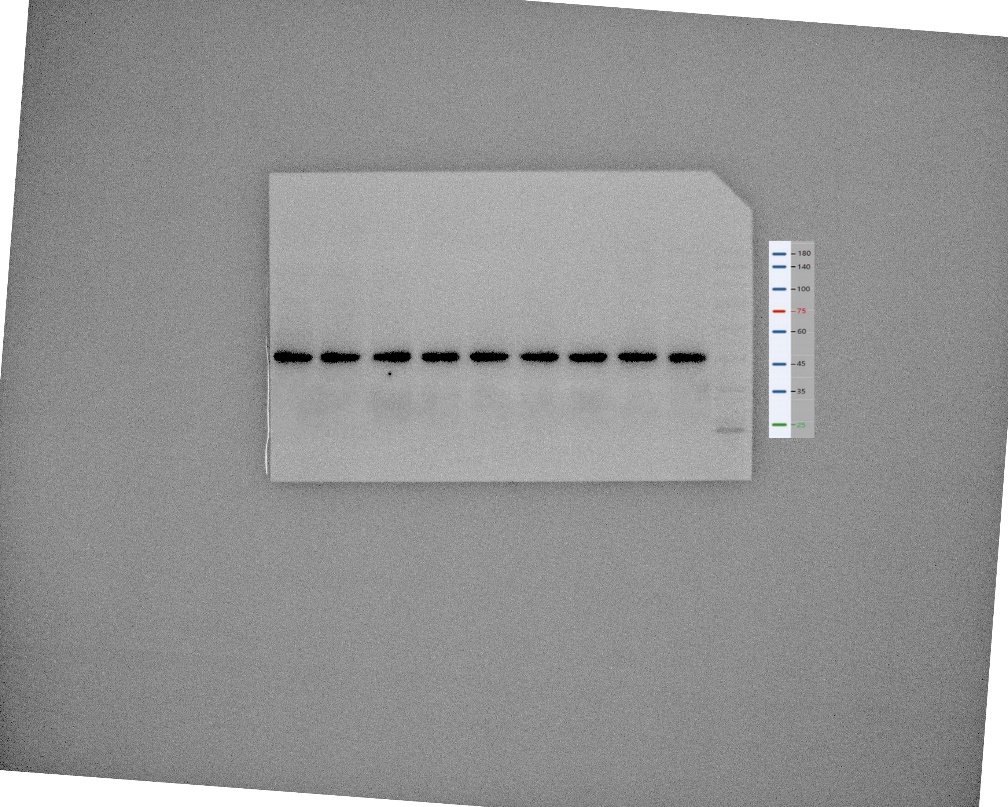


β-actin

Supplement: Supplementary file 1 — Original Data File [file 41420_2024_1855_MOESM1_ESM.docx]
